# Associations among multidomain lifestyles, chronic diseases, and dementia in older adults: a cross-sectional analysis of a cohort study

**DOI:** 10.3389/fnagi.2023.1200671

**Published:** 2023-08-04

**Authors:** Jing-jing Zhang, Zhao-xia Wu, Wei Tan, Dan Liu, Gui-rong Cheng, Lang Xu, Fei-fei Hu, Yan Zeng

**Affiliations:** ^1^Geriatric Hospital Affiliated to Wuhan University of Science and Technology, Wuhan, China; ^2^Brain Science and Advanced Technology Institute, Wuhan University of Science and Technology, Wuhan, China; ^3^School of Public Health, Wuhan University of Science and Technology, Wuhan, China; ^4^Wuchang Hospital Affiliated to Wuhan University of Science and Technology, Wuhan, China

**Keywords:** dementia, chronic disease, community-dwelling older adults, synergistic effect, lifestyle

## Abstract

**Background:**

Unhealthy lifestyles and chronic diseases are commonly seen and treatable factors in older adults and are both associated with dementia. However, the synergistic effect of the interaction of lifestyles and chronic diseases on dementia is unknown.

**Methods:**

We determined independent associations of multidomain lifestyles and chronic diseases (cerebrovascular disease, diabetes, and hypertension) with dementia and examined their synergistic impact on dementia among older adults. The data were drawn from the Hubei Memory and Aging Cohort Study. We created a summary score of six factors for multidomain lifestyles. Dementia was diagnosed according to the Diagnostic and Statistical Manual of Mental Disorders IV. Logistic regression and multiple correspondence analyses were used to explore the relationships among multidomain lifestyles, chronic diseases, and dementia. A sensitivity analysis was performed to minimize the interference of reverse causality and potential confounders.

**Results:**

Independent associations with dementia were found in unhealthy (OR = 1.90, 95% CI: 1.38–2.61) and intermediate healthy lifestyles (OR, 3.29, 2.32–4.68), hypertension (OR, 1.21, 1.01–1.46), diabetes (OR, 1.30, 1.04–1.63), and cerebrovascular disease (OR, 1.39, 1.12–1.72). Interactions of diabetes (*p* = 0.004), hypertension (*p* = 0.004), and lifestyles were significant, suggesting a combined impact on dementia. Sensitivity analysis supported the strong association among multidomain lifestyles, chronic diseases, and dementia prevalence.

**Conclusion:**

An unhealthy lifestyle was associated with a higher prevalence of dementia, regardless of whether the participants had chronic diseases; however, this association was stronger in individuals with chronic diseases. Multidomain lifestyles and chronic diseases may have an enhanced impact on dementia.

## 1. Introduction

With an aging population and dramatic changes in lifestyles, the rising incidence of dementia has become an urgent public health problem (Chan et al., 2013). Dementia affects > 57 million people globally, which is set to increase to 152 million by 2050 (GBD 2019 Dementia Forecasting Collaborators, 2022). Studies have shown that altering 12 modifiable risk factors could delay or prevent the onset of 40% of dementia cases worldwide (Livingston et al., 2020). However, these studies have more often revealed individual or a couple of life behaviors (Baumgart et al., 2015; Smith and Blumenthal, 2016); hence, interventions targeting several risk factors have been trialed in older participants (Ngandu et al., 2015, 2021). Few researchers have investigated the combined effects of multidomain lifestyles on the prevalence of dementia, particularly in people with chronic diseases. Considering that dementia has complex pathogenesis and causes, some lifestyle factors may not be sufficient to individually influence dementia-related brain changes, and complex interactions between these factors may exert enhanced effects (Weuve et al., 2018).

The global increase in the prevalence of type 2 diabetes (Gregg et al., 2016), hypertension (Sun et al., 2022), and cerebrovascular diseases (Hao et al., 2021) has been well documented. Adults with type 2 diabetes, hypertension, and cerebrovascular disease are at increased risk of developing dementia (Biessels et al., 2008). These chronic diseases are significantly associated with multidomain lifestyles (Boehme et al., 2017; Altobelli et al., 2020; Ozemek et al., 2020; Hoshide et al., 2023; Zyriax and Windler, 2023) and an increased risk of dementia in the same direction (Janson et al., 2004; Brands et al., 2005; Biessels et al., 2006; Yang et al., 2017; Ou et al., 2020). Recent studies have shown that a more active life can mitigate the deleterious impact of chronic diseases on dementia (Marseglia et al., 2020; Wang et al., 2020). Therefore, it is crucial to understand complicated relationships among multidomain lifestyles, chronic diseases, and dementia in the context of the new era and to develop intensive prevention strategies that are in line with the current reality of social life.

In response to the urgent need to clarify the relationship among multidomain lifestyles, chronic diseases, and dementia, this study investigated the independent associations of multidomain lifestyles and chronic diseases with dementia and examined whether these factors interact with each other creating greater risks of dementia in older adults.

## 2. Materials and methods

### 2.1. Study participants

We used baseline data from the Hubei Memory and Aging Cohort Study (ChiCTR1800019164), a longitudinal cohort study characterized by the collection of multidimensional risk factors and cognitive aging trajectories in a large community-based old population. Detailed information about the cohort was described previously (Li et al., 2022). The sample included in the study comprised participants aged 65 years and older. We excluded persons with dementia diagnosed by a physician, serious physical illness, psychiatric illness, severe visual and hearing impairment, severely impaired ability to perform activities of daily living (ADL), and missing information. A total of 8,221 residents were included in this study. All procedures were performed in accordance with the basic principles of the Declaration of Helsinki and were approved by the Ethics Committee of the Medical School of Wuhan University of Science and Technology. All participants provided written informed consent.

### 2.2. Assessment of multidomain lifestyle

Lifestyle data were obtained from the participants’ self-reports of their past experiences. In the main analysis, lifestyle was determined by summing the scores for six factors: smoking, alcohol consumption, dietary pattern, physical activity, cognitive activity, and waist circumference. Healthy status received a score of 1, whereas the opposite received a score of 0. Healthy smoking status was defined as never smoking; healthy drinking status was defined as moderate (and never) drinking (no more than 28 g of alcohol per day for men and no more than 14 g of alcohol per day for women) (Lloyd-Jones et al., 2010); healthy eating pattern was defined as eating a balanced diet that includes nutrient-rich foods, such as vegetables, fruits, whole grains, lean proteins, and healthy fats (Bojang and Manchana, 2023); regular physical activity was defined as at least 150 min of moderate to vigorous activity per week (U.S. Department of Health and Human Services and U.S. Department of Agriculture, 2015); regular cognitive activity was defined as at least one cognitive activity per day (reading books, reading newspapers, playing chess, playing cards, playing games, speculating on stocks, etc.); and healthy waist circumference was defined as <80 cm for women and <85 cm for men. Lifestyle scores ranged from 0 to 6, with higher scores representing healthier lifestyles, and were divided into three groups: unhealthy (0–2), intermediate healthy (3–4), and healthy (5–6).

### 2.3. Cognition evaluation and diagnoses

This study used a standardized cognitive assessment battery to test the cognitive status of the participants. These tests included (1) the Mini-Mental State Examination (MMSE) (Creavin et al., 2016) and the Montreal Cognitive Assessment-Basic (MoCA-BC) (Cao et al., 2012) to assess global cognition; (2) the auditory verbal learning test (An et al., 2018), shape trail tests A and B, forward and backward conditions of the digit span test, Boston naming test (Miebach et al., 2019) and animal fluency test, and clock-drawing test with 5 scales to assess memory, executive ability, attention, language ability, and visuospatial function, respectively.

Two neuropsychologists with expertise in dementia confirmed the diagnosis of MCI and dementia based on Petersen’s MCI judgment criteria and the Diagnostic and Statistical Manual of Mental Disorders, 5th edition (DSM-V) criteria. The diagnostic criteria for MCI were as follows: (1) the presence of cognitive impairment reported or found by the patient, informant, and/or clinician; (2) the presence of objective evidence of impairment in at least one domain of cognitive functioning not limited to memory; (3) retention of independent functional abilities, though instrumental abilities might have been slightly impaired [assessed by clinical dementia rating and ADL scales]; and (4) no diagnosis of dementia (assessed by DSM-V) (Petersen, 2004). The diagnostic criteria for dementia were as follows: (1) previous normal cognition, (2) acquired cognitive decline or abnormal mental behavior, (3) affected work ability or daily life, and (4) cannot be explained by delirium or other mental illness (American Psychiatric Association [APA], 2000).

### 2.4. Other measures

Other measures included sociodemographic characteristics, past medical history, family history, personal life history, physical examination, and laboratory tests. The Geriatric Depression Scale was used to assess depression in older adults over the past 6 months (Shin et al., 2019), and ADL and instrumental ADL scales were used to assess the participants’ ability and disability in daily living (Lawton and Brody, 1969).

### 2.5. Statistical analysis

Continuous variables were found to be non-normally distributed using the Kolmogorov-Smirnov test; therefore, data for this component were expressed as median (interquartile range). Categorical variables were expressed as numbers (percentages). The differences between participants with and without dementia were analyzed using the Mann-Whitney U test and chi-square test. Univariate logistic regression was used to assess the strength of the association between the correlates and dementia. Multivariate adjusted models were used to explore the relationship between univariate lifestyle factors and dementia. In adjusted model, we adjusted for residence area, age, sex, education level, hypertension, diabetes, and cerebrovascular disease. We then used logistic regression models to assess the association among lifestyle classification, chronic diseases, and dementia separately. We performed partial correlation analysis to analyze the relationships among multidomain lifestyles, chronic diseases, and dementia. Subgroup analyses were performed to explore whether the association between lifestyles and dementia differed based on health status. Multiple correspondence analyses was performed to further explore the multiple relationships among multidomain lifestyles, chronic diseases, and dementia.

Finally, seven sensitivity analyses were conducted to minimize confounding by reverse causality and potential confounders: (1) conducted Spearman’s correlation analysis to analyze the linear relationship between lifestyle scores and cognitive function scores; (2) excluded participants who showed significant loss of life skills based on ADL scores to rule out the effect of loss of life skills on lifestyle changes; (3) replaced moderate alcohol consumption with never drinking, as the effect of alcohol consumption on dementia is controversial; (4) redefined healthy diet as a balanced diet (balanced meat and vegetables), as some participants with diabetes reported concerns about exacerbating their condition by consuming fruits; (5) included good sleep status, i.e., defined as no insomnia; (6) further analyzed the relationship between lifestyle classification and dementia only in MCI and dementia participants; and (7) performed multiple correspondence analysis to analyze the multiple relationship between lifestyles, chronic diseases, and cognitive status. All *p*-values were bilateral, and the results were considered statistically significant at *p* < 0.05. SPSS version 26.0 was used for all statistical analyses.

## 3. Results

### 3.1. Sociodemographic characteristics

Table 1 demonstrates the characteristics of the participants with respect to sociodemographics, chronic diseases, and lifestyle factors. A total of 8,221 eligible participants aged 65 years and older, including 3,772 men and 4,449 women, were included. They were 3,164 rural and 5,057 urban participants. The proportions of participants aged 65–69, 70–74, 75–79, and ≥80 years were 41.9, 29.8, 15.7, and 12.6%, respectively. Further, 595 (7.2%) participants were diagnosed with dementia, and the prevalence of hypertension, diabetes mellitus, and cerebrovascular disease was 66.2, 16.6, and 17.5%, respectively. Participants with dementia were more likely to be rural (*p* < 0.001), older (*p* < 0.001), female (*p* < 0.001), and less educated (*p* < 0.001). Participants with dementia had higher rates of hypertension (*p* = 0.036) and cerebrovascular disease (*p* = 0.006) than those without dementia. Although some risk factors related to an unhealthy lifestyle were equally present in both participants with and without dementia, those without dementia were more inclined to have a healthy lifestyle (*p* < 0.001), which included a healthy diet pattern (*p* < 0.001), physical activity (*p* < 0.001), and cognitive activity (*p* < 0.001). No significant differences were noted in other factors between the groups.

**TABLE 1:** Sociodemographic characteristics, chronic diseases, and lifestyle factors of the participants.

Characteristics	Total, *n* (%)	Non-dementia, *n* (%)	Dementia, *n* (%)	*p*-value
Number of participants	8221	7626 (92.8)	595 (7.2)	
Sex (female)	4449 (54.1)	4082 (53.5)	367 (61.7)	<0.001**
Age (years), median (IQR)	71 (67, 75)	70 (67, 75)	76 (71, 81)	<0.001**
Age groups, in years				<0.001**
65–69	3441 (41.9)	3327 (43.6)	114 (19.2)	
70–74	2448 (29.8)	2282 (29.9)	166 (27.9)	
75–79	1292 (15.7)	1168 (15.3)	124 (20.8)	
≥80	1040 (12.6)	849 (11.1)	191 (32.1)	
Education (years), median (IQR)	9 (3, 12)	9 (3, 12)	2 (0, 8)	<0.001**
Education level				<0.001**
Primary school or below	3425 (41.7)	3043 (39.9)	382 (64.2)	
Junior high school	1955 (23.8)	1850 (24.3)	105 (17.6)	
Senior high school	1483 (18.0)	1426 (18.7)	57 (9.6)	
University or above	1358 (16.5)	1307 (17.1)	51 (8.6)	
Residential area (rural)	3164 (38.5)	2791 (36.3)	373 (62.7)	<0.001**
Diabetes	1367 (16.6)	1257 (16.5)	110 (18.5)	0.206
Hypertension	5440 (66.2)	5023 (65.9)	417 (70.1)	0.036*
Cerebrovascular disease	1440 (17.5)	1311 (17.2)	129 (21.7)	0.006**
Never smoking	6215 (75.6)	5743 (75.3)	472 (79.3)	0.028*
Moderate drinking	6546 (79.6)	6077 (79.7)	469 (78.8)	0.614
Healthy diet	2340 (28.5)	2271 (29.8)	69 (11.6)	<0.001**
Physical activity	6653 (80.9)	6282 (82.4)	371 (62.4)	<0.001**
Cognitive activity	4914 (59.8)	4734 (62.1)	180 (30.3)	<0.001**
Healthy waist circumference	2405 (29.3)	2232 (29.3)	173 (29.1)	0.921
Lifestyle category				<0.001**
Healthy	1799 (21.9)	1750 (22.9)	49 (8.2)	
Intermediate	4784 (58.2)	4447 (58.3)	337 (56.6)	
Unhealthy	1638 (19.9)	1429 (18.7)	209 (35.1)	

*p < 0.05; **p < 0.01.

### 3.2. Correlations among multidomain lifestyle, chronic diseases, and dementia

First, we assessed the strength of the individual association of six lifestyle factors, i.e., smoking, moderate alcohol consumption, dietary patterns, physical activity, cognitive activity, and waist circumference with dementia using univariate logistic regression (Table 2). Then, we built the multivariate adjusted models and found that having a healthy diet (OR = 0.50, 95% CI: 0.38–0.67, *p* < 0.001), regular physical activity (OR = 0.53, 95% CI: 0.44–0.63, *p* < 0.001), and cognitive activity (OR = 0.40, 95% CI: 0.32–0.48, *p* < 0.001) were significantly associated with a lower prevalence of dementia.

**TABLE 2:** Univariate analysis of lifestyle factors associated with dementia.

Characteristics	Unadjusted model			Adjusted model 1			Adjusted model 2		
	Odds ratio	95% CI	*p*-value	Odds ratio	95% CI	*p*-value	Odds ratio	95% CI	*p*-value
Never smoking	1.26	1.03, 1.55	0.028*	0.78	0.61, 1.01	0.055	0.78	0.61, 1.01	0.056
No	1 (Ref)			1 (Ref)			1 (Ref)		
Moderate drinking	0.95	0.77, 1.16	0.614	0.84	0.67, 1.06	0.141	0.83	0.66, 1.04	0.112
No	1 (Ref)			1 (Ref)			1 (Ref)		
Healthy diet	0.31	0.24, 0.40	<0.001**	0.51	0.39, 0.67	<0.001**	0.50	0.38, 0.67	<0.001**
No	1 (Ref)			1 (Ref)			1 (Ref)		
Physical activity	0.35	0.30, 0.42	<0.001**	0.52	0.43, 0.62	<0.001**	0.53	0.44, 0.63	<0.001**
No	1 (Ref)			1 (Ref)			1 (Ref)		
Cognitive activity	0.27	0.22, 0.32	<0.001**	0.39	0.32, 0.48	<0.001**	0.40	0.32, 0.48	<0.001**
No	1 (Ref)			1 (Ref)			1 (Ref)		
Healthy waist circumference	0.99	0.82, 1.19	0.921	1.11	0.92, 1.35	0.292	1.16	0.95, 1.41	0.146
No	1 (Ref)			1 (Ref)			1 (Ref)		

*p < 0.05; **p < 0.01.

Model 1 adjusted for area, age, sex, and education level.

Model 2 adjusted for area, age, sex, education level, hypertension, diabetes and cerebrovascular disease.

Second, we evaluated the strength of the association of individual health conditions with dementia (Table 3) and found that participants with diabetes had a 30% increased risk of dementia compared to that of those without diabetes (OR = 1.30, 95% CI: 1.04–1.63, *p* < 0.05). Moreover, cerebrovascular disease was positively associated with dementia (OR = 1.39, 95% CI: 1.12–1.72, *p* < 0.01). We further assessed the association of multidomain lifestyles with dementia and found that participants with an intermediate healthy lifestyle (OR = 1.90, 95% CI: 1.38–2.61, *p* < 0.001) were less associated with dementia than those with an unhealthy lifestyle (OR = 3.29, 95% CI: 2.32–4.68, *p* < 0.001).

**TABLE 3:** Associations of dementia with lifestyle and chronic diseases.

Group	Unadjusted model			Adjusted model 1			Adjusted model 2		
	Odds ratio	95% CI	*p*-value	Odds ratio	95% CI	*p*-value	Odds ratio	95% CI	*p*-value
**Lifestyle category**									
Healthy	1 (Ref)			1 (Ref)			1 (Ref)		
Intermediate	2.71	2.00, 3.67	<0.001**	1.92	1.40, 2.64	<0.001**	1.90	1.38, 2.61	<0.001**
Unhealthy	5.22	3.80, 7.19	<0.001**	3.36	2.36, 4.77	<0.001**	3.29	2.32, 4.68	<0.001**
Diabetes	1.15	0.93, 1.43	0.206	1.33	1.06, 1.66	0.014*	1.30	1.04, 1.63	0.023*
No	1 (Ref)			1 (Ref)			1 (Ref)		
Hypertension	1.21	1.01, 1.46	0.036*	1.03	0.85, 1.24	0.789	0.99	0.81, 1.19	0.880
No	1 (Ref)			1 (Ref)			1 (Ref)		
Cerebrovascular disease	1.33	1.09, 1.64	0.006**	1.41	1.14, 1.74	0.002**	1.39	1.12, 1.72	0.003**
No	1 (Ref)			1 (Ref)			1 (Ref)		

*p < 0.05; **p < 0.01.

Model 1 adjusted for area, age, sex, and education level.

Model 2 adjusted for area, age, sex, education level, hypertension, diabetes and cerebrovascular disease.

**FIGURE 1:**
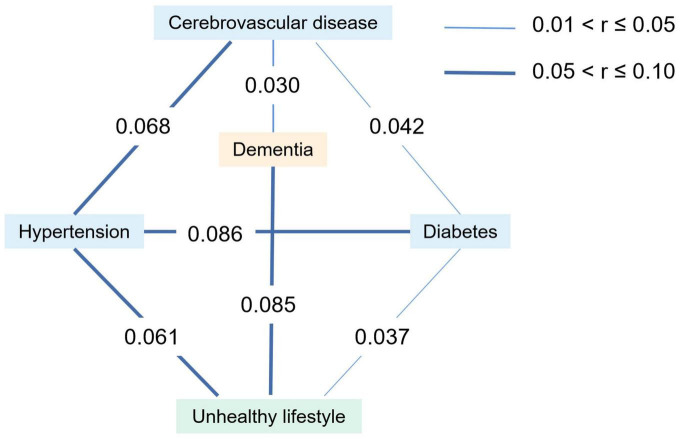
Correlation analyses between lifestyle categories, chronic diseases and dementia. The figure shows the statistically significant correlation coefficient (r) among unhealthy lifestyle, hypertension, diabetes, cerebrovascular disease and dementia. The thin correlation line indicates 0.01 < r ≤ 0.05, and the thick correlation line indicates 0.05 < r ≤ 0.10.

Third, our partial correlation analysis for multidomain lifestyles, chronic diseases, and dementia showed that both cerebrovascular disease (*r* = 0.030, *p* = 0.007) and unhealthy lifestyle (*r* = 0.085, *p* < 0.001) were independently associated with dementia. Additionally, hypertension (*r* = 0.061, *p* < 0.001) and diabetes (*r* = 0.037, *p* = 0.001) were positively associated with unhealthy lifestyle. Among the three chronic diseases, the associations between hypertension, diabetes, and cerebrovascular disease were statistically significant (r _Hypertension–Diabetes_ = 0.086, *p* < 0.001; r _Hypertension–Cerebrovascular disease_ = 0.068, *p* < 0.001; r _Diabetes–Cerebrovascular disease_ = 0.042, *p* < 0.001) (Figure 1).

**FIGURE 2:**
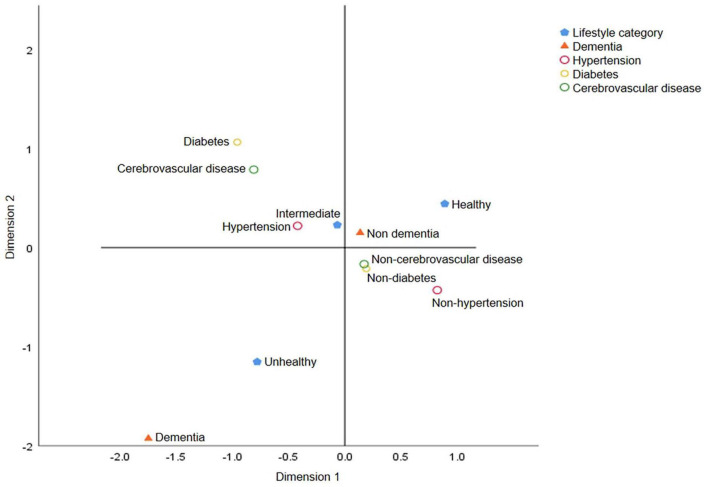
Multiple correspondence analysis charts for lifestyle categories, chronic diseases and dementia. The graph reflects the degree of association among lifestyle categories (healthy, intermediate, and unhealthy lifestyle), chronic diseases and dementia. The closer the category points are, the greater the correlation is. Unhealthy lifestyle and dementia are located in the same quadrant and far away from the center of the circle, indicating that these two points have distinct characteristics and are quite different from other points.

Fourth, to determine the possible interaction effect of lifestyles and chronic diseases on dementia prevalence, we conducted interaction tests and found interactions of hypertension (*p* = 0.004) and diabetes (*p* = 0.010) but not of cerebrovascular disease (*p* = 0.817) with lifestyles. We performed subgroup analyses using multifactorial logistic regression and found that among participants without diabetes, those with intermediate healthy (OR = 1.56, 95% CI: 1.11–2.19, *p* = 0.010) and unhealthy lifestyles (OR = 2.70, 95% CI: 1.86–3.93, *p* < 0.001) had a stronger association with dementia prevalence than those with a healthy lifestyle. A similar association was noted between lifestyle and the prevalence of dementia in participants with diabetes (OR _intermediate_ = 7.14, 95% CI: 2.18–23.34, *p* = 0.001; OR _unhealthy_ = 12.25, 95% CI: 3.56–42.14, *p* < 0.001). The model that included all participants demonstrated that a healthy lifestyle was associated with a lower prevalence of dementia, regardless of whether the participants had diabetes (Table 4). As for participants with hypertension and cerebrovascular disease, both intermediate healthy and unhealthy lifestyles were found to be positively associated with the prevalence of dementia, unlike healthy lifestyle (Supplementary Table 1). To more clearly and intuitively determine the complex relationship among different lifestyles, the prevalence of three chronic diseases, and different cognitive states, we conducted a multiple correspondence analysis and found that participants with an unhealthy lifestyle were more likely to have chronic diseases and dementia (Figure 2).

**TABLE 4:** Relationship between lifestyle and dementia in group with diabetes or not.

Diabetes		Unadjusted model			Adjusted model		
		Odds ratio	95% CI	*p*-value	Odds ratio	95% CI	*p*-value
**In separate model**							
No	Healthy	1 (Ref)			1 (Ref)		
	Intermediate	2.33	1.69, 3.20	<0.001**	1.56	1.11, 2.19	0.010*
	Unhealthy	4.67	3.34, 6.52	<0.001**	2.70	1.86, 3.93	<0.001**
Yes	Healthy	1 (Ref)			1 (Ref)		
	Intermediate	8.31	2.59, 26.59	<0.001**	7.14	2.18, 23.34	0.001**
	Unhealthy	13.70	4.16, 45.07	<0.001**	12.25	3.56, 42.14	<0.001**
**In one model**							
No	Healthy	1 (Ref)			1 (Ref)		
	Intermediate	2.33	1.69, 3.20	<0.001**	1.59	1.14, 2.22	0.007**
	Unhealthy	4.67	3.34, 6.52	<0.001**	2.77	1.92, 4.00	<0.001**
Yes	Healthy	0.36	0.11, 1.18	0.092	0.34	0.10, 1.10	0.071
	Intermediate	3.02	2.06, 4.42	<0.001**	2.24	1.51, 3.32	<0.001**
	Unhealthy	4.99	3.16, 7.88	<0.001**	3.83	2.35, 6.24	<0.001**

*p < 0.05; **p < 0.01.

Model adjusted for area, age, sex, education level, hypertension, diabetes, and cerebrovascular disease.

Finally, in the sensitivity analysis, we explored the correlation between lifestyle scores and the MMSE, MoCA, and ADL scores. We found a positive correlation between lifestyle and cognitive functioning scores, independently of chronic diseases. A significant negative correlation was observed between multidomain lifestyle and function ability scores, and this association was similar in participants with (rs = −0.19, *p* < 0.001) and without chronic diseases (rs = −0.20, *p* < 0.001) (Supplementary Table 2). We excluded participants with severely impaired function ability and analyzed the association between multidomain lifestyle classification and dementia prevalence and found no substantial change. An unhealthy lifestyle was significantly associated with a higher rate of dementia in both groups with (OR = 2.95, 95% CI: 1.86–4.69, *p* = 0.001) and without chronic diseases (OR = 3.02, 95% CI: 1.32–6.95, *p* = 0.009) (Supplementary Table 3). After redefining moderate alcohol consumption as never drinking, reanalysis showed that the association of unhealthy (OR _No chronic diseases_ = 3.48, 95% CI: 1.60–7.56, *p* = 0.002; OR _Chronic diseases_ = 3.02, 95% CI: 2.04–4.49, *p* < 0.001) and intermediate healthy lifestyles (OR _No chronic diseases_ = 2.34, 95% CI: 1.17–4.72, *p* = 0.017; OR _Chronic diseases_ = 1.67, 95% CI: 1.16–2.40, *p* = 0.005) with higher dementia prevalence was statistically significant in different health conditions (Supplementary Table 4). The association in the results remained significant after removing the criterion of daily fruit consumption from the healthy diet (OR _No chronic diseases_ = 3.64, 95% CI: 1.93–6.89, *p* < 0.001; OR _Chronic diseases_ = 2.95, 95% CI: 2.09–4.18, *p* < 0.001; Supplementary Table 5). Furthermore, this association was statistically significant with the addition of sleep status to lifestyle (OR _No chronic diseases_ = 4.13, 95% CI: 2.18–7.80, *p* < 0.001; OR _Chronic diseases_ = 3.19, 95% CI: 2.29–4.46, *p* < 0.001; Supplementary Table 6). When analysis of the sample included with MCI and dementia only, we found that an unhealthier lifestyle was continuously associated with a higher prevalence of dementia (OR _No chronic diseases–Unhealthy_ = 4.97, 95% CI: 2.18–11.33, *p* < 0.001; OR _Chronic diseases–Unhealthy_ = 2.89, 95% CI: 1.90–4.39, *p* < 0.001; Supplementary Table 7). Additionally, an intermediate lifestyle was associated with three chronic diseases and MCI. Finally, multiple correspondence analyses showed a strong association between unhealthy lifestyle and dementia and chronic diseases (Supplementary Figure 1).

## 4. Discussion

We determined independent associations of six lifestyle factors and three chronic diseases with dementia and demonstrated their enhanced combined impact on dementia among older adults. Although some individual lifestyle factors were not strongly associated with dementia prevalence, a comprehensive assessment of multidomain lifestyles showed a strong correlation with dementia prevalence; this correlation was stronger in patients with some chronic diseases. Our study suggests there may be a complex relationship among lifestyle, chronic diseases, and dementia. Although there are substantial evidences that healthy lifestyles may reduce the risk of cognitive decline and dementia (Lee et al., 2018; Amakye et al., 2019; Marseglia et al., 2019; Du Preez et al., 2021; Nabe-Nielsen et al., 2021), few trials have attempted to reveal the role of lifestyles in preventing dementia (Lee et al., 2014; Ngandu et al., 2015; Gregory et al., 2016; Andrieu et al., 2017). Our analysis found that some individual lifestyle variables were not significantly associated with dementia prevalence, whereas the combination of lifestyle factors had a reinforcing effect. In addition, most studies did not include patients with chronic diseases, a population at high risk of dementia. In this study, lifestyle was defined as a holistic concept to study the combined effect of multidomain lifestyle on dementia prevalence, with a specific focus on whether there was a similar association between individuals with and without chronic diseases. To minimize the impact of possible reverse causality of lifestyle changes due to dementia, all analyses were repeated after excluding participants with severely impaired life ability at baseline survey. The association between lifestyles and dementia prevalence among the participants was not altered, regardless of chronic diseases, suggesting that our findings are unlikely to be attributed to reverse causality.

Other lifestyle factors that were not included in the six comprehensive ones may also have an impact on the development of dementia. Therefore, we redefined some lifestyle factors, and our results showed a consistent association between lifestyle and dementia. In addition, we performed Spearman’s correlation analysis to present the continuous correlation among lifestyle scores, cognitive function, and life ability scores. The results further showed that an unhealthier lifestyle was related to worse cognitive abilities.

Previous studies have confirmed that a healthy lifestyle is associated with a lower risk of cardiovascular disease and a healthier physical condition (Marmot, 2020; Zhang et al., 2021) as well as a lower risk of dementia (Anstey et al., 2009; Fletcher et al., 2013; Blondell et al., 2014; Mozaffarian, 2016). For example, never smokers and former smokers may have a healthier diet, such as eating more vegetables and fruits (Kawada, 2004), and a lower risk of cardiovascular disease (Dobson et al., 1991), both of which may be beneficial for delaying or reversing the pathological process of dementia (Sabia et al., 2008). Middle-aged and older men have a higher prevalence of cardiovascular disease and mortality, and thus the association between smoking, alcohol consumption, and dementia may be underestimated (Chêne et al., 2015; Du Preez et al., 2021). The mechanism of the relationship between lifestyle factors and dementia is not fully understood. Vascular, inflammation, oxidative stress, neurotoxicity, and psychosocial processes may play a role in the association between lifestyle related factors and pathological process of dementia (Dening et al., 2020), such as smoking (Dobson et al., 1991; Kawachi et al., 1993, 1994), drinking (Zahr et al., 2011). A healthy dietary pattern plays an important role in maintaining nerve membrane integrity and upregulating neurotrophic factors (Coley et al., 2015). In addition, the gut-brain axis is beneficial for maintaining brain health and the progression of neurological diseases (Carabotti et al., 2015). There is evidence that physical activity can increase cognitive reserve, which is the brain’s ability to resist in response to injury (Fratiglioni et al., 2004). Physical activity not only increases brain volume (Rovio et al., 2010; Ho et al., 2011) and levels of brain-derived neurotrophic factor (Komulainen et al., 2008), but also improves high blood pressure and elevated blood sugar levels, which are risk factors for dementia. This is beneficial to cardiovascular health and reduces metabolic risk (Ho et al., 2011). In addition, physical activity can also effectively relieve psychological stress (Fratiglioni et al., 2004). Cognitive activity can increase cognitive reserve and brain volume, which is similar to physical activity (Liu et al., 2012). Cardiovascular risk factors play a potential role in the association between waist circumference and cognitive function. Central obesity is associated with cardiovascular risk factors such as high blood pressure and high cholesterol (Tchernof and Després, 2013), and it is also an important risk factor for cardiovascular disease and T2D (Chang et al., 2012). These cardiovascular risk factors and cardiovascular disease are significantly associated with dementia (Whitmer et al., 2005; Kloppenborg et al., 2008). Moreover, other lifestyle factors and genetics may also play a role in their association. As a complex and multi-factorial disease, dementia may require multidomain comprehensive research on many related factors and disease mechanisms. Therefore, consideration of multidomain lifestyles can lead to a comprehensive and objective understanding of the correlation between lifestyle and dementia. One study has shown that the combination of modifiable risk factors is related to the risk of dementia (Peters et al., 2019); the healthy levels of many risk factors are controversial. Therefore, we reclassified the health criteria of some risk factors to explore the relationship among multidomain lifestyle, chronic diseases, and dementia under different criteria.

Previous studies have shown that the association between chronic diseases and dementia may be mediated by cardiovascular risk factors, education, socioeconomic status, and lifestyle factors (Twig et al., 2014), with equally complex interactions among these factors. Therefore, it is necessary to comprehensively examine the effects of multidomain lifestyles on cognitive function in patients with chronic diseases. Our study suggests that participants with diabetes and a healthy lifestyle were more strongly associated with a lower prevalence of dementia than those without diabetes. This indicates that the effect of healthy lifestyles does not seem to be affected by diabetes. Moreover, hypertension interacted with lifestyle in our study, suggesting that hypertension and diabetes have an interactive effect with lifestyle on the prevalence of dementia. The interaction effect between cerebrovascular disease and lifestyle was not significant; however, an unhealthy lifestyle was strongly associated with the prevalence of dementia, suggesting that lifestyle affects the prevalence of dementia through a pathway independent of cerebrovascular disease. Accelerated aging and age-related cerebrovascular diseases are associated with many pathophysiological processes. Regardless of the risk factors in previous studies, the complex vascular phenotype changes caused by aging make the brain prone to vascular cognitive impairment and dementia (Ungvari et al., 2010). Studies have shown that atherosclerosis, the main vascular lesion of vascular cognitive impairment disorders, may be associated with aging and persistent low-grade inflammation (Franceschi et al., 2000; Csiszar et al., 2008; Nilsson, 2015; O’Brien and Thomas, 2015). These inflammatory factors increase with age, independent of known risk factors (Bruunsgaard et al., 2000; Miles et al., 2008). This suggests that cerebrovascular disease and lifestyle-related risk factors may independently influence cognitive function. In the correspondence analysis, we found that people who endorsed healthier lifestyle was more likely to have better cognitive status and no chronic diseases, whereas those with an unhealthy lifestyle were more strongly associated with dementia than hypertension, diabetes, and cerebrovascular disease. Overall, our study suggests that people without chronic diseases should adapt to a healthy lifestyle to reduce their risk of developing dementia. Especially, people with chronic diseases were encouraged to reduce the likelihood of developing dementia in the future by re-establishing a healthy lifestyle. This is because people with chronic diseases can have more benefit in preventing dementia by having a healthy lifestyle than those without chronic diseases.

The present study has some limitations. First, although the response rate in this study was relatively high, we cannot exclude the possibility of selection bias among the participants. We excluded participants who were unable to participate in the survey for physical reasons; however, we could not confirm whether their physical conditions were associated with dementia. Second, the information on lifestyle included in our study was mainly through self-reports, which may have led to subjective cognitive errors. Although we validated these self-reports using health records from health centers, there may still be an impact on the true correlation. Third, the included indicators of lifestyle-related factors were limited and reflected only some aspects of lifestyle; other lifestyle factors may also affect dementia prevalence. The lifestyle scores in this study were derived from the sum of the scores of lifestyle-related factors. We assumed that these lifestyle factors have the same effect on physical status; however, this may have obscured the real interactions between lifestyle factors. Fourth, there may be potential confounding factors that interfere with our results, such as other physical conditions and socioeconomic status. Participants may have had serious medical conditions at baseline that were not covered in the survey, affecting their lifestyle behaviors and socioeconomic status. Fifth, since the present data were derived from an observational study, it was impossible to make clear causal inferences about the findings. Although our multivariate adjusted model analysis and sensitivity analysis produced results that were consistent with those of the main analysis, reverse causality and residual confounding may have existed.

## 5. Conclusion

Despite these limitations, based on a large size sample, sufficient amount of high-quality data, multivariate adjusted model analyses, and a series of sensitivity analyses, this study highlights the fact that an unhealthier multidomain lifestyle is associated with a higher prevalence of dementia, and this association is stronger in people with chronic diseases such as diabetes. Based on this, we suggest that people prioritize lifestyle modifications to prevent dementia. Particularly, people with chronic diseases who are at a high risk of dementia may gain greater preventive effects.

## Data availability statement

The raw data supporting the conclusions of this article will be made available by the authors, without undue reservation.

## Ethics statement

All procedures involving human participants in this study were in accordance with the basic principles of the Declaration of Helsinki. The study was approved by the Ethics Committee of the Medical School of Wuhan University of Science and Technology (protocol code: 201845; approved on October 22, 2018).

## Author contributions

J-jZ: conceptualization (supporting), formal analysis (lead), investigation (equal), methodology (supporting), software (lead), validation (equal), writing–original draft preparation (supporting), and writing–review and editing (supporting). Z-xW and WT: resources (lead). DL: supervision (equal) and validation (equal). G-rC: data curation (lead) and supervision (equal). LX: funding acquisition (equal). F-fH: investigation (equal). YZ: conceptualization (lead), funding acquisition (equal), methodology (lead), project administration (lead), supervision (equal), validation (equal), writing–original draft preparation (lead), and writing–review and editing (lead). All authors contributed to the article and approved the submitted version.
