# Supplementary material for: Associations among multidomain lifestyles, chronic diseases, and dementia in older adults: a cross-sectional analysis of a cohort study
Source: Front Aging Neurosci. 2023 Aug 4;15:1200671. doi: 10.3389/fnagi.2023.1200671 (PMC10438989; doi:10.3389/fnagi.2023.1200671)
Supplement: Supplementary file 1 [file Table_1.docx]

Supplementary Material

Associations among multidomain lifestyles, chronic diseases, and dementia in older adults: A cross-sectional analysis of a cohort study

Jing-jing Zhang^#^, Zhao-xia Wu^#^, Wei Tan, Dan Liu, Gui-rong Cheng, Lang Xu, Fei-fei Hu and Yan Zeng^*^

**^#^ Joint first authors:** Jing-jing Zhang, Zhao-xia Wu

*** Correspondence:** Yan Zeng: zengyan68@wust.edu.cn

**S1 TABLE** Relationship between lifestyle categories and dementia in hypertension and cerebrovascular disease or not.

| **In separate model** | | | | |  |  | | | |
| --- | --- | --- | --- | --- | --- | --- | --- | --- | --- |
|  | | Hypertension | | |  | Cerebrovascular disease | | | |
|  |  | Odds ratio | 95% CI | p value |  | Odds ratio | 95% CI |  | p value |
| No | Healthy | 1 (Ref) |  |  |  | 1 (Ref) |  |  |  |
|  | Intermediate | 2.20 | 1.21, 3.99 | 0.010* |  | 1.99 | 1.38, 2.87 |  | < 0.001** |
|  | Unhealthy | 3.52 | 1.81, 6.84 | < 0.001** |  | 3.19 | 2.13, 4.77 |  | < 0.001** |
| Yes | Healthy | 1 (Ref) |  |  |  | 1 (Ref) |  |  |  |
|  | Intermediate | 1.69 | 1.16, 2.48 | 0.007** |  | 1.63 | 0.84, 3.19 |  | 0.151 |
|  | Unhealthy | 3.10 | 2.05, 4.69 | < 0.001** |  | 3.85 | 1.86, 7.99 |  | < 0.001** |
| **In one model** | | | | |  |  | | | |
|  | | Hypertension | | |  | Cerebrovascular disease | | | |
|  |  | Odds ratio | 95% CI | p value |  | Odds ratio | 95% CI |  | p value |
| No | Healthy | 1 (Ref) |  |  |  | 1 (Ref) |  |  |  |
|  | Intermediate | 2.43 | 1.37, 4.32 | 0.003** |  | 2.01 | 1.40, 2.89 |  | < 0.001** |
|  | Unhealthy | 3.72 | 1.99, 6.95 | <0.001** |  | 3.15 | 2.12, 4.68 |  | < 0.001** |
| Yes | Healthy | 1.33 | 0.71, 2.50 | 0.374 |  | 1.43 | 0.73, 2.80 |  | 0.298 |
|  | Intermediate | 2.23 | 1.28, 3.89 | 0.005** |  | 2.29 | 1.50, 3.51 |  | < 0.001** |
|  | Unhealthy | 4.07 | 2.29, 7.26 | <0.001** |  | 5.79 | 3.62, 9.25 |  | < 0.001** |

*p < 0.05; **p < 0.01

Model adjusted for area, age, sex, education level, hypertension, diabetes and cerebrovascular disease.

**S2 TABLE** Relationship between lifestyle score and cognitive function.

| Population | MMSE score | |  | MoCA score | |  | ADL score | |
| --- | --- | --- | --- | --- | --- | --- | --- | --- |
|  | rs | p value |  | rs | p value |  | rs | p value |
| Total | 0.31 | < 0.001** |  | 0.25 | < 0.001** |  | -0.20 | < 0.001** |
| No chronic diseases | 0.27 | < 0.001** |  | 0.29 | < 0.001** |  | -0.20 | < 0.001** |
| chronic diseases | 0.33 | < 0.001** |  | 0.23 | < 0.001** |  | -0.19 | < 0.001** |

*p < 0.05; **p < 0.01

**S3 TABLE** Relationship between lifestyle categories and dementia in chronic diseases or not after excluding individuals with ADL scores showed severe loss of activities of daily living.

| Comorbidities |  | Unadjusted model | | |  | Adjusted model | | |
| --- | --- | --- | --- | --- | --- | --- | --- | --- |
|  |  | Odds ratio | 95% CI | p value |  | Odds ratio | 95% CI | p value |
| No | Healthy | 1 (Ref) |  |  |  | 1 (Ref) |  |  |
|  | Intermediate | 3.67 | 1.82, 7.41 | < 0.001** |  | 2.15 | 1.02, 4.51 | 0.043* |
|  | Unhealthy | 6.19 | 2.92, 13.11 | < 0.001** |  | 3.02 | 1.32, 6.95 | 0.009** |
| Yes | Healthy | 1 (Ref) |  |  |  | 1 (Ref) |  |  |
|  | Intermediate | 2.67 | 1.79, 3.99 | < 0.001** |  | 1.99 | 1.31, 3.02 | < 0.001** |
|  | Unhealthy | 4.37 | 2.86, 6.67 | < 0.001** |  | 2.95 | 1.86, 4.69 | 0.001** |

*p < 0.05; **p < 0.01

Model adjusted for area, age, sex and education level.

**S4 TABLE** Relationship between lifestyle categories and dementia in chronic diseases or not after replacing moderate alcohol consumption with never drinking.

| Comorbidities |  | Unadjusted model | | |  | Adjusted model | | |
| --- | --- | --- | --- | --- | --- | --- | --- | --- |
|  |  | Odds ratio | 95% CI | p value |  | Odds ratio | 95% CI | p value |
| No | Healthy | 1 (Ref) |  |  |  | 1 (Ref) |  |  |
|  | Intermediate | 4.02 | 2.07, 7.80 | < 0.001** |  | 2.34 | 1.17, 4.72 | 0.017* |
|  | Unhealthy | 6.63 | 3.29, 13.34 | < 0.001** |  | 3.48 | 1.60, 7.56 | 0.002** |
| Yes | Healthy | 1 (Ref) |  |  |  | 1 (Ref) |  |  |
|  | Intermediate | 2.26 | 1.60, 3.18 | < 0.001** |  | 1.67 | 1.16, 2.40 | 0.005** |
|  | Unhealthy | 4.27 | 2.99, 6.11 | < 0.001** |  | 3.02 | 2.04, 4.49 | <0.001** |

*p < 0.05; **p < 0.01

Model adjusted for area, age, sex and education level.

**S5 TABLE** Relationship between lifestyle categories and dementia in chronic diseases or not after defining a healthy diet as a balanced diet with meat and vegetables.

| Comorbidities |  | Unadjusted model | | |  | Adjusted model | | |
| --- | --- | --- | --- | --- | --- | --- | --- | --- |
|  |  | Odds ratio | 95% CI | p value |  | Odds ratio | 95% CI | p value |
| No | Healthy | 1 (Ref) |  |  |  | 1 (Ref) |  |  |
|  | Intermediate | 2.82 | 1.79, 4.45 | <0.001** |  | 1.72 | 1.05, 2.81 | 0.031* |
|  | Unhealthy | 6.17 | 3.52, 10.83 | <0.001** |  | 3.64 | 1.93, 6.89 | < 0.001** |
| Yes | Healthy | 1 (Ref) |  |  |  | 1 (Ref) |  |  |
|  | Intermediate | 2.04 | 1.59, 2.63 | <0.001** |  | 1.64 | 1.26, 2.14 | < 0.001** |
|  | Unhealthy | 3.62 | 2.65, 4.95 | <0.001** |  | 2.95 | 2.09, 4.18 | < 0.001** |

*p < 0.05; **p < 0.01

Model adjusted for area, age, sex and education level.

**S6 TABLE** Relationship between lifestyle categories and dementia in chronic diseases or not after adding sleep status in the lifestyle score.

| Comorbidities |  | Unadjusted model | | |  | Adjusted model | | |
| --- | --- | --- | --- | --- | --- | --- | --- | --- |
|  |  | Odds ratio | 95% CI | p value |  | Odds ratio | 95% CI | p value |
| No | Healthy | 1 (Ref) |  |  |  | 1 (Ref) |  |  |
|  | Intermediate | 3.96 | 2.44, 6.45 | < 0.001** |  | 2.62 | 1.55, 4.40 | < 0.001** |
|  | Unhealthy | 7.55 | 4.27, 13.34 | < 0.001** |  | 4.13 | 2.18, 7.80 | < 0.001** |
| Yes | Healthy | 1 (Ref) |  |  |  | 1 (Ref) |  |  |
|  | Intermediate | 2.27 | 1.75, 2.93 | < 0.001** |  | 1.79 | 1.36, 2.34 | < 0.001** |
|  | Unhealthy | 4.31 | 3.19, 5.83 | < 0.001** |  | 3.19 | 2.29, 4.46 | < 0.001** |

*p < 0.05; **p < 0.01

Model adjusted for area, age, sex and education level.

**S7 TABLE** Relationship between lifestyle categories and dementia in chronic diseases or not after including individuals with MCI or dementia.

| Comorbidities |  | Unadjusted model | | |  | Adjusted model | | |
| --- | --- | --- | --- | --- | --- | --- | --- | --- |
|  |  | Odds ratio | 95% CI | p value |  | Odds ratio | 95% CI | p value |
| No | Healthy | 1 (Ref) |  |  |  | 1 (Ref) |  |  |
|  | Intermediate | 3,14 | 1.58, 6.26 | 0.001** |  | 2.29 | 1.11, 4.74 | 0.026* |
|  | Unhealthy | 6.09 | 2.88, 12.85 | < 0.001** |  | 4.97 | 2.18, 11.33 | < 0.001** |
| Yes | Healthy | 1 (Ref) |  |  |  | 1 (Ref) |  |  |
|  | Intermediate | 2.05 | 1.43, 2.95 | < 0.001** |  | 1.67 | 1.14, 2.45 | 0.008** |
|  | Unhealthy | 3.68 | 2.51, 5.41 | < 0.001** |  | 2.89 | 1.90, 4.39 | < 0.001** |

*p < 0.05; **p < 0.01

Model adjusted for area, age, sex and education level.


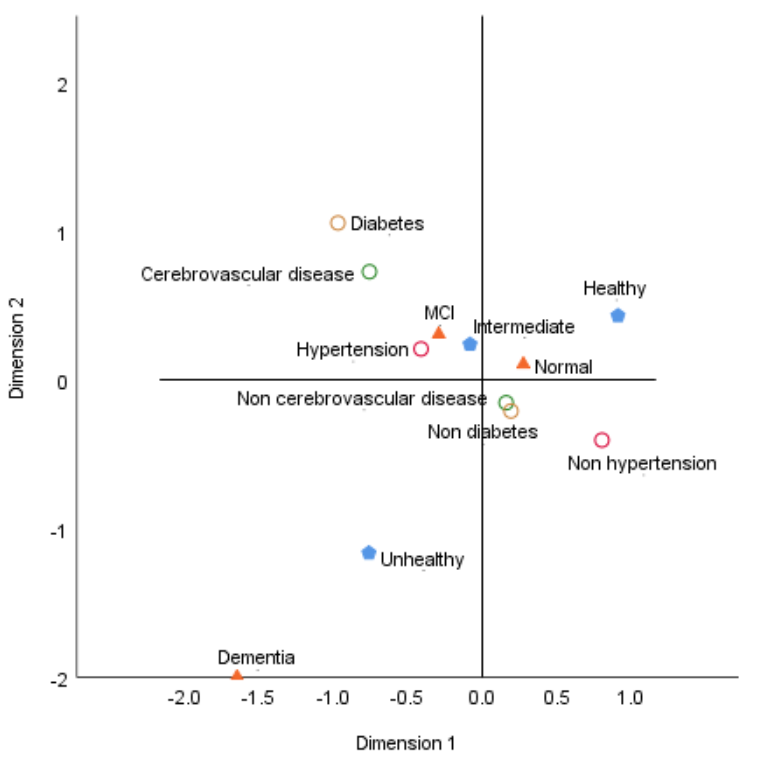


**FIGURE S1** Corelations between lifestyle categories, chronic diseases and cognitive status by multiple correspondence analysis charts.
